# Supplementary material for: The pitfalls of using Gaussian Process Regression for normative modeling
Source: PLoS One. 2021 Sep 15;16(9):e0252108. doi: 10.1371/journal.pone.0252108 (PMC8443061; doi:10.1371/journal.pone.0252108)
Supplement: S1 Appendix — All datasets are generated from the same Python script, which contains the code for analysis as well; Eq S1 is a detailed derivation for Eq 11; S1 Fig and S1 Table are results for modified Datasets 3-4. (PDF) [file pone.0252108.s001.pdf]

Appendix

Data/code availability

The results presented in the main text and appendix are generated with Python 3.8.7 and scikit-learn 0.24.0. Although we have not tested, it should work with other version of Python and package as well. The code for this paper is available at: `https://github.com/nidaye1999/normative-model-GPR`.

Derivation for Eq. 11

We present a detailed derivation of Eq. 11 here. By substituting $\boldsymbol{\Sigma}_{\text{train}}^{-1}\boldsymbol{Z}(\boldsymbol{X})$ with $\boldsymbol{U}\boldsymbol{\Sigma}\boldsymbol{V}^T$, we can get

$$
\begin{aligned}
&\Sigma_*^2(\boldsymbol{x}_*)\\
=&\boldsymbol{z}(\boldsymbol{x}_*)\boldsymbol{z}(\boldsymbol{x}_*)^T+\sigma_{\text{test}}^2-\boldsymbol{z}(\boldsymbol{x}_*)\boldsymbol{Z}(\boldsymbol{X})^T\left[\boldsymbol{Z}(\boldsymbol{X})\boldsymbol{Z}(\boldsymbol{X})^T+\boldsymbol{\Sigma}_{\text{train}}^2\right]^{-1}\boldsymbol{Z}(\boldsymbol{X})\boldsymbol{z}(\boldsymbol{x}_*)^T\\
=&\boldsymbol{z}(\boldsymbol{x}_*)\boldsymbol{z}(\boldsymbol{x}_*)^T+\sigma_{\text{test}}^2-\boldsymbol{z}(\boldsymbol{x}_*)\boldsymbol{Z}(\boldsymbol{X})^T\left\{\boldsymbol{\Sigma}_{\text{train}}\left[\left(\boldsymbol{\Sigma}_{\text{train}}^{-1}\boldsymbol{Z}(\boldsymbol{X})\right)\left(\boldsymbol{\Sigma}_{\text{train}}^{-1}\boldsymbol{Z}(\boldsymbol{X})\right)^T+\boldsymbol{I}\right]\boldsymbol{\Sigma}_{\text{train}}\right\}^{-1}\boldsymbol{Z}(\boldsymbol{X})\boldsymbol{z}(\boldsymbol{x}_*)^T\\
=&\boldsymbol{z}(\boldsymbol{x}_*)\boldsymbol{z}(\boldsymbol{x}_*)^T+\sigma_{\text{test}}^2-\boldsymbol{z}(\boldsymbol{x}_*)\boldsymbol{Z}(\boldsymbol{X})^T\boldsymbol{\Sigma}_{\text{train}}^{-1}\left[\left(\boldsymbol{\Sigma}_{\text{train}}^{-1}\boldsymbol{Z}(\boldsymbol{X})\right)\left(\boldsymbol{\Sigma}_{\text{train}}^{-1}\boldsymbol{Z}(\boldsymbol{X})\right)^T+\boldsymbol{I}\right]^{-1}\boldsymbol{\Sigma}_{\text{train}}^{-1}\boldsymbol{Z}(\boldsymbol{X})\boldsymbol{z}(\boldsymbol{x}_*)^T\\
=&\boldsymbol{z}(\boldsymbol{x}_*)\boldsymbol{z}(\boldsymbol{x}_*)^T+\sigma_{\text{test}}^2-\boldsymbol{z}(\boldsymbol{x}_*)\left(\boldsymbol{\Sigma}_{\text{train}}^{-1}\boldsymbol{Z}(\boldsymbol{X})\right)^T\left[\left(\boldsymbol{\Sigma}_{\text{train}}^{-1}\boldsymbol{Z}(\boldsymbol{X})\right)\left(\boldsymbol{\Sigma}_{\text{train}}^{-1}\boldsymbol{Z}(\boldsymbol{X})\right)^T+\boldsymbol{I}\right]^{-1}\left(\boldsymbol{\Sigma}_{\text{train}}^{-1}\boldsymbol{Z}(\boldsymbol{X})\right)\boldsymbol{z}(\boldsymbol{x}_*)^T\\
=&\boldsymbol{z}(\boldsymbol{x}_*)\boldsymbol{z}(\boldsymbol{x}_*)^T+\sigma_{\text{test}}^2-\boldsymbol{z}(\boldsymbol{x}_*)\left(\boldsymbol{U}\boldsymbol{\Sigma}\boldsymbol{V}^T\right)^T\left[\left(\boldsymbol{U}\boldsymbol{\Sigma}\boldsymbol{V}^T\right)\left(\boldsymbol{U}\boldsymbol{\Sigma}\boldsymbol{V}^T\right)^T+\boldsymbol{I}\right]^{-1}\left(\boldsymbol{U}\boldsymbol{\Sigma}\boldsymbol{V}^T\right)\boldsymbol{z}(\boldsymbol{x}_*)^T\\
=&\boldsymbol{z}(\boldsymbol{x}_*)\boldsymbol{z}(\boldsymbol{x}_*)^T+\sigma_{\text{test}}^2-\boldsymbol{z}(\boldsymbol{x}_*)\boldsymbol{V}\boldsymbol{\Sigma}^T\boldsymbol{U}^T\left[\boldsymbol{U}\boldsymbol{\Sigma}\boldsymbol{V}^T\boldsymbol{V}\boldsymbol{\Sigma}^T\boldsymbol{U}^T+\boldsymbol{I}\right]^{-1}\boldsymbol{U}\boldsymbol{\Sigma}\boldsymbol{V}^T\boldsymbol{z}(\boldsymbol{x}_*)^T\\
=&\boldsymbol{z}(\boldsymbol{x}_*)\boldsymbol{z}(\boldsymbol{x}_*)^T+\sigma_{\text{test}}^2-\boldsymbol{z}(\boldsymbol{x}_*)\boldsymbol{V}\boldsymbol{\Sigma}^T\boldsymbol{U}^T\left[\boldsymbol{U}\boldsymbol{\Sigma}\boldsymbol{\Sigma}^T\boldsymbol{U}^T+\boldsymbol{U}\boldsymbol{I}\boldsymbol{U}^T\right]^{-1}\boldsymbol{U}\boldsymbol{\Sigma}\boldsymbol{V}^T\boldsymbol{z}(\boldsymbol{x}_*)^T\\
=&\boldsymbol{z}(\boldsymbol{x}_*)\boldsymbol{z}(\boldsymbol{x}_*)^T+\sigma_{\text{test}}^2-\boldsymbol{z}(\boldsymbol{x}_*)\boldsymbol{V}\boldsymbol{\Sigma}^T\boldsymbol{U}^T\left[\boldsymbol{U}\left(\boldsymbol{\Sigma}\boldsymbol{\Sigma}^T+\boldsymbol{I}\right)\boldsymbol{U}^T\right]^{-1}\boldsymbol{U}\boldsymbol{\Sigma}\boldsymbol{V}^T\boldsymbol{z}(\boldsymbol{x}_*)^T\\
=&\boldsymbol{z}(\boldsymbol{x}_*)\boldsymbol{z}(\boldsymbol{x}_*)^T+\sigma_{\text{test}}^2-\boldsymbol{z}(\boldsymbol{x}_*)\boldsymbol{V}\boldsymbol{\Sigma}^T\boldsymbol{U}^T\boldsymbol{U}^{T^{-1}}\left(\boldsymbol{\Sigma}\boldsymbol{\Sigma}^T+\boldsymbol{I}\right)^{-1}\boldsymbol{U}^{-1}\boldsymbol{U}\boldsymbol{\Sigma}\boldsymbol{V}^T\boldsymbol{z}(\boldsymbol{x}_*)^T\\
=&\boldsymbol{z}(\boldsymbol{x}_*)\boldsymbol{z}(\boldsymbol{x}_*)^T+\sigma_{\text{test}}^2-\boldsymbol{z}(\boldsymbol{x}_*)\boldsymbol{V}\boldsymbol{\Sigma}^T\left(\boldsymbol{\Sigma}\boldsymbol{\Sigma}^T+\boldsymbol{I}\right)^{-1}\boldsymbol{\Sigma}\boldsymbol{V}^T\boldsymbol{z}(\boldsymbol{x}_*)^T\\
=&\underbrace{\boldsymbol{z}(\boldsymbol{x}_*)\boldsymbol{V}\left[\boldsymbol{I}-\boldsymbol{\Sigma}^T\left(\boldsymbol{\Sigma}\boldsymbol{\Sigma}^T+\boldsymbol{I}\right)^{-1}\boldsymbol{\Sigma}\right]\boldsymbol{V}^T\boldsymbol{z}(\boldsymbol{x}_*)^T}_{\text{quadratic term}}+\underbrace{\sigma_{\text{test}}^2}_{\text{constant}},
\end{aligned}
\tag{S1}
$$

where $\boldsymbol{\Sigma}$ is a diagonal matrix and the elements on the diagonal are the singular values of $\boldsymbol{\Sigma}_{\text{train}}^{-1}\boldsymbol{Z}(\boldsymbol{X})$, $\boldsymbol{U}$ and $\boldsymbol{V}$ are orthogonal.

GPR with complex datasets

A quadratic function $y = x^2$ is utilized as the true reference model in the following case, while the other parameters are exactly same to Datasets 3-4.

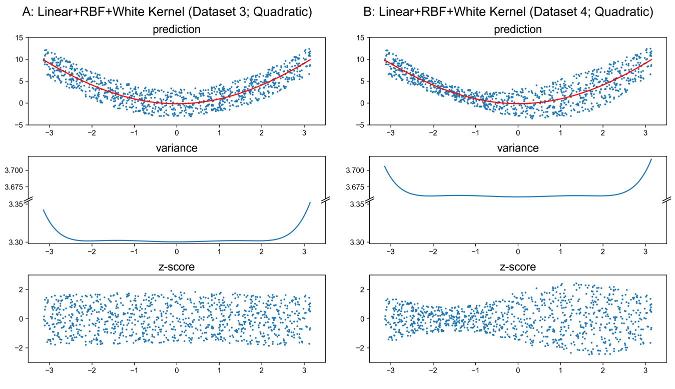

(a) (b)

S1 Fig. GPR with Hybrid Kernel on Modified Datasets 3-4.

S1 **Table. Optimized Hyper-parameters for Hybrid Kernel on Modified Datasets 3-4**.

	w_{linear}	w_{RBF}	l	σ^2_{noise}	$Var\,(y - y_{\text{reference}})$
**Dataset 3**	1.69e-4	1.73e3	7.25	3.29	3.28
**Dataset 4**	1.69e-4	1.86e3	7.39	3.65	3.64
