# Supplementary material for: The pitfalls of using Gaussian Process Regression for normative modeling
Source: PLoS One. 2021 Sep 15;16(9):e0252108. doi: 10.1371/journal.pone.0252108 (PMC8443061; doi:10.1371/journal.pone.0252108)
Supplement: Attachment — Submitted filename: Response to Reviewers.pdf [file pone.0252108.s002.pdf]

Dear academic editor and reviewer,

Response to academic editor:

1. ***Comment:*** *Please review your reference list to ensure that it is complete and correct. If you have cited papers that have been retracted, please include the rationale for doing so in the manuscript text, or remove these references and replace them with relevant current references. Any changes to the reference list should be mentioned in the rebuttal letter that accompanies your revised manuscript. If you need to cite a retracted article, indicate the article's retracted status in the References list and also include a citation and full reference for the retraction notice.*

   Response: The modifications in the reference list are highlighted in the pink color. The DOIs are added to all the references when applicable. No cited paper/material has been retracted.

2. ***Comment:*** *Please ensure that your manuscript meets PLOS ONE's style requirements, including those for file naming. The PLOS ONE style templates can be found at*

   `https://journals.plos.org/plosone/s/file?id=wjVg/PLOSOne_formatting_sample_main_body.pdf` *and*

   `https://journals.plos.org/plosone/s/file?id=ba62/PLOSOne_formatting_sample_title_authors_affiliations.pdf`

   Response: The footnotes in the previous version are moved into the main text and appendix. The corresponding modifications in the main text are highlighted in the gray color.

3. ***Comment:*** *Thank you for stating the following in the Acknowledgments Section of your manuscript:*

   [Also, special thanks to Laureate Institute for Brain Research and the University of Tulsa for supporting this research.]

   We note that you have provided funding information that is not currently declared in your Funding Statement. However, funding information should not appear in the Acknowledgments section or other areas of your manuscript. We will only publish funding information present in the Funding Statement section of the online submission form.

   Please remove any funding-related text from the manuscript and let us know how you would like to update your Funding Statement. Currently, your Funding Statement reads as follows:

   [This research was supported by the Laureate Institute for Brain Research and the National Institute of General Medical Sciences (P20GM121312, MP, RK)]

   Please include your amended statements within your cover letter; we will change the online submission form on your behalf.

   Response: Part of the acknowledgment has been modified and highlighted in the green color. We don't need to update the Funding Statement.

4. ***Comment:*** *We note that Figure 1 in your submission contain copyrighted images. All PLOS content is published under the Creative Commons Attribution License (CC BY 4.0), which means that the manuscript, images, and Supporting Information files will be freely available online, and any third party is permitted to access, download, copy, distribute, and use these materials in any way, even commercially, with proper attribution. For more information, see our copyright guidelines:* `http://journals.plos.org/plosone/s/licenses-and-copyright`.

   Response: We got the permission for using that plot in the paper before the submission. We tried to contact the World Health Organization again to fill out the Content Permission Form but not get any response yet. Since the permission was sent to us via email, will that email work as an approval for using Figure 1 in this paper?

Response to Reviewer #1:

1. ***Comment:*** *In Row 64, "where* $\boldsymbol{\Sigma}_{train}^2$ *and* $\boldsymbol{\Sigma}_{test}^2$ *are two diagonal matrices that represent the variance of observation noise in training and testing sets, and the diagonal elements equal* σ_{noise}^2*". Are all diagonal elements equal* σ_{noise}^2*?*

   Response: These two matrices represent the noises in observations for training and testing sets, respectively. Because we assume that each training/testing observation noise is independent and sampled from one Gaussian distribution $\mathcal{N}\left(0, \sigma_{\text{noise}}^2\right)$ in GPR, the diagonal elements of these two square matrices are all identical and equal to the variance of the Gaussian noise for observation σ_{noise}^2, while all the other off-diagonal elements are 0s.

   The corresponding sentence has been modified and highlighted in yellow as "where $\boldsymbol{\Sigma}_{\text{train}}^2$ and $\boldsymbol{\Sigma}_{\text{test}}^2$ are two square diagonal matrices that represent the variance of observation noise in training and testing sets, and all diagonal elements of $\boldsymbol{\Sigma}_{\text{train}}^2$ and $\boldsymbol{\Sigma}_{\text{test}}^2$ are identical and equal to σ_{noise}^2".

2. ***Comment:*** *The limited results are not comprehensive enough. The experimental results of other kernel methods should be reported for a fair comparison.*

   Response: We used a rigorous mathematical derivation within a general form to prove our conclusion in this paper, which applies to all the kernels. We chose linear and RBF kernels with one-dimensional input data as examples, because they are the simplest and most widely-used kernels and we can visualize the results, which would help the readers to have a better understanding about the properties of the uncertainty estimated by GPR.

   We also added the descriptions and results of other two RBF-based but more complex kernels (Matérn kernel and Rational-Quadratic kernel) into the revised paper. The corresponding contents are highlighted in yellow as well.

3. ***Comment:*** *The pictures shown in this paper are too vague, I suggest the author redraw them.*

   Response: We also notice this issue. All plots we uploaded are high quality, but we do not know why they become vague in the downloaded pdf file. We will work with the journal to fix that.
